# Predictors of Hospitalization for Patients Presenting to Emergency Department with COVID-19 Infection

**DOI:** 10.3390/jcm14020413

**Published:** 2025-01-10

**Authors:** Alhareth Alsagban, Amteshwar Singh, Anurima Baidya, Monika Dalal, Waseem Khaliq

**Affiliations:** 1 Department of Medicine, Johns Hopkins University School of Medicine, Baltimore, MD 21224, USA; 2 Department of International Health, Johns Hopkins Bloomberg School of Public Health, Baltimore, MD 21205, USA; 3 Department of Epidemiology, Johns Hopkins Bloomberg School of Public Health, Baltimore, MD 21205, USA

**Keywords:** COVID-19, hospitalization, mortality, emergency department

## Abstract

**Background**: Predictors of morbidity and mortality in hospitalized COVID-19 patients have been extensively studied. However, comparative analyses of predictors for hospitalization versus discharge from the emergency department remain limited. **Methods**: This retrospective study evaluated predictors of hospitalization among adults (≥18 years) presenting to the emergency department with COVID-19 infection between 1 March 2020 and 15 June 2020. Data were obtained from electronic health records across five hospitals within the Johns Hopkins Health System, encompassing 2513 beds. Multivariable logistic regression models were employed to assess the association between sociodemographic characteristics, clinical symptoms, and comorbidities with hospitalization. **Results**: Of the 2767 patients presenting to the emergency department, 1678 (61%) were hospitalized, while 1089 (39%) were discharged from the emergency department. Hospitalized patients were older (mean age 61.8 years, SD 18), more likely to be African American and White, non-Hispanic, unemployed or on disability, medically insured, had access to primary care, and presented on weekends. Smoking status, alcohol use, and higher comorbidity burden (mean age-adjusted Charlson Comorbidity Index > 3) were also more prevalent with hospitalization. Dyspnea was a prominent clinical feature among hospitalized patients. After adjusting for sociodemographic and clinical risk factors, significant predictors of hospitalization included health insurance (OR 3.44; 95% CI: 1.98–6), having a primary care (OR 1.85; 95% CI: 1.33–2.59), presentation from a non-home locale (OR 4.04; 95% CI: 1.93–8.47), age-adjusted CCI > 3 (OR 1.72; 95% CI: 1.11–2.68), dyspnea (OR 2.22; 95% CI: 1.56–3.17), neutrophil-to-lymphocyte ratio ≥ 3 (OR 2.17; 95% CI: 1.54–3.06), and an abnormal chest radiograph findings (OR 6.17; 95% CI: 4.40–8.66). Interestingly, obesity, defined as a BMI ≥ 30 kg/m^2^ (OR 0.45; 95% CI: 0.32–0.64), and the presence of fever (OR 0.64; 95% CI: 0.43–0.95) were found to be associated with a decreased likelihood of hospitalization. **Conclusions**: Future studies are warranted to further explore predictors of COVID-19 hospitalization, with particular focus on the implications of weekend presentations and the paradoxical relationship of obesity with COVID-19 health outcomes. These findings could inform the development of triage models to enhance preparedness for future pandemics.

## 1. Introduction

The COVID-19 pandemic has profoundly affected global health, with more than 760 million confirmed cases and 6.9 million deaths recorded worldwide since December 2019 [1]. In the first year of the pandemic alone, weekly visits to the emergency department (ED) rose to 6.8%, with weekly mortality as high as 23,364 per 100,000 persons in the United States (U.S.) in 2020 [2]. By the end of 2020, the cumulative COVID-19 hospitalization rate was as high as 179.9 per 100,000 persons, which continued to rise to 510.7 in 2021 [2]. These staggering numbers highlight the critical need for effective strategies to manage COVID-19-related health issues, especially within emergency departments (ED), where initial patient evaluations occur.

Research has focused on identifying clinical predictors that can effectively stratify the risk of hospitalization for patients presenting to the emergency department (ED) with COVID-19 infection [3]. For instance, older age, frailty, the presence of underlying comorbid conditions (e.g., cardiovascular disease, chronic kidney disease, hypertension, etc.), and laboratory markers (e.g., elevated d-dimer, low albumin, etc.) have been associated with poor health outcomes in COVID-19 infected patients [4,5,6,7,8,9,10]. However, there remains a notable gap in the literature regarding predictors of hospitalization for patients presenting with COVID-19 infection to the ED. Understanding these hospitalization predictors is crucial for improving triage decision-making, resource management, throughput models, and patient outcomes. Improved identification of patients at high risk for hospitalization can optimize healthcare resource allocation, thereby alleviating the burden on healthcare systems already strained by the pandemic’s ongoing challenges.

This observational retrospective study aims to identify factors predictive of hospitalization for patients presenting to the emergency department (ED) with COVID-19 infection during the initial surge of the pandemic in 2020 [11]. We further aim to identify the most common diagnoses associated with these hospitalizations.

## 2. Methods

### 2.1. Study Design and Sample Size

We performed a retrospective review of medical records in a single healthcare system. The descriptive study consisted of all adult patients with COVID-19 infection aged ≥ 18 years who were evaluated in the Johns Hopkins Health System emergency departments between 1 March 2020 and 15 June 2020 during the pre-COVID-vaccine era. The data for the study were collected using electronic medical records from five hospitals comprising 2513 beds in the Johns Hopkins Health System (Johns Hopkins Hospital, Baltimore, Maryland; Bayview Medical Center, Baltimore, Maryland; Howard County General Hospital, Columbia, Maryland; Suburban Hospital, Bethesda, Maryland; and Sibley Hospital, Washington, DC, USA). COVID-19 was defined as the detection of SARS-CoV-2 from any nucleic acid test combined with an International Statistical Classification of Diseases and Related Health Problems, Tenth Revision (ICD-10) code indicating the presence of symptomatic disease [12]. Patients were excluded if aged < 18 years, pregnant, and presented for psychiatry evaluation or admission. In addition, patients who presented to the ED multiple times during the study period were only included during their first encounter. The Institutional Review Board of the health care system, in addition to each hospital board, approved the study protocol.

There were 3192 emergency department encounters eligible for study enrollment, of which 425 encounters were excluded according to the above-mentioned criteria. Therefore, the final study population consisted of 2767 patients evaluated at the emergency departments of the Johns Hopkins Health care system.

### 2.2. Study Protocol, Variables, and Measures

A careful review of the medical records was performed to determine the diagnosis of COVID-19 infection, ED length of stay, weekend encounter (Saturday or Sunday), and type of encounter (emergency department vs. hospital admission). Sociodemographic information (age, race, gender, ethnicity, marital status, and employment) and health behavior (smoking status, alcohol use, body mass index) at the time of encounter were extracted during an extensive review of the medical records. Access to health care was assessed by health insurance status and having a primary care provider. We also evaluated the chronic disease burden by collecting information about several medical comorbidities, including those needed for the Charlson comorbidity index (CCI), a method of categorizing comorbidities by mortality risk status based on the International Classification of Diseases (ICD) diagnosis codes [13]. Clinical symptoms, vital statistics (blood pressure, heart rate, oxygen level, respiratory rate, and temperature), and laboratory and imaging workups were extracted by a detailed review of medical documentation at the time of initial emergency department encounter within the health care system. Similarly, clinical examination and workup during hospitalization were also ascertained for the hospitalized patients. Primary diagnoses at the time of admission and during discharge were extracted and matched to determine the final diagnosis. Where these diagnoses differed, the discharging diagnosis was considered the final diagnosis. A detailed abstraction sheet was developed for all the above-mentioned information. Two independent data abstractors entered the data separately using an abstraction sheet. Whenever there was a discrepancy, the data were rechecked by a third person to confirm the correct information.

### 2.3. Statistical Analysis/Methods

Patient characteristics are presented as proportions and means. Unpaired t-tests and Chi-square tests were used to compare study population characteristics, clinical symptoms, laboratory and imaging workup, and medical comorbidities by hospitalization vs. ED discharge status. *T*-tests and Chi-square tests determined significance at *p*-value ≤ 0.05. Logistic regression models were used to assess risk factors associated with hospitalization. Multivariable logistic regression models assessed associations between sociodemographic, clinical symptoms, and comorbidity variables thought to predict hospitalization. We chose predictors that were thought to explain hospitalization based on clinical relevance and the prior literature. To assess multicollinearity among predictor variables, we calculated the correlation matrix and used the Variance Inflation Factor (VIF). Each individual predictor variable had a VIF value of less than 2, indicating low multicollinearity. The mean VIF for all predictors combined was 1.22, further confirming the absence of significant multicollinearity in the model. We used logistic regression models for analyses to predict the odds of medical hospitalization for patients with COVID-19 infection. Logistic regression models were used for sociodemographics, clinical symptoms, laboratory and imaging workup, and medical comorbidity burden models separately. The resultant models were then adjusted for all possible risk factors for the final analyses. The study data were analyzed using Stata statistical software (StataCorp LP, College Station, TX, USA, Version 16.1).

## 3. Results

Of 2767 patients presenting to the emergency department, 1678 (61%) were admitted to the hospital and 1089 (39%) were discharged from the emergency department. The mean age of the study population was 55 years (SD 19); 34% were African American, and 33% reported Hispanic ethnicity. Table 1 delineates the similarities and differences of the study population based on their disposition from the ED (ED discharges vs. hospitalization). The population characteristics varied between the two groups; e.g., the hospitalized group was more likely to be from an older age group, White or African American, non-Hispanic in ethnicity, unemployed or with a disability, medically insured, had a primary care provider and likely to present to ED over the weekend. The mean ED length of stay for the hospitalized group was approximately 6.5 h compared to ED discharges (3.25 h). Hospitalized patients were more likely to report current or prior cigarette smoking and alcohol use. In addition, hospitalized patients also had a higher comorbidity burden (mean age-adjusted Charlson Comorbidity Index > 3) and presented to the ED from non-home locale (i.e., nursing homes, inpatient rehabilitation centers, group homes, adult daycare centers, or with unhoused status).

Table 2 highlights clinical symptoms, vital signs, laboratory and imaging workup at presentation to ED stratified by ED discharge and hospitalization. Fever, dry cough, and myalgia were the most frequently reported clinical symptoms among patients discharged from the ED. In contrast, shortness of breath was the most prominent clinical feature among hospitalized patients. Neutrophil to lymphocyte count ratio (NLR) of ≥3 and abnormal read on chest X-ray (CXR) were the most prominent workup findings among hospitalized patients. Table 3 shows the frequency of clinical signs, laboratory workup, and imaging studies among patients admitted to the hospital.

In partially adjusted, bivariate analysis, 9 of the 14 sociodemographic variables and 6 of the 13 clinical and laboratory variables were associated with hospitalization among patients presenting to the ED with COVID-19 infection (Table 4). After adjusting for sociodemographic, clinical, laboratory, and imaging workup variables that could potentially influence hospitalization, the odds of hospitalization were 3.44 times higher for patients with health insurance (OR 3.44; 95% CI: 1.98–6) as compared to uninsured patients, 1.85 times higher for patients utilizing a primary care (OR 1.85; 95% CI: 1.33–2.59) as compared to patients without a primary care provider, 4.04 times higher for patients presentation from a non-home locale (OR 4.04; 95% CI: 1.93–8.47) compared to arriving to ED from home, 1.72 time higher among patients with age-adjusted CCI > 3 (OR 1.72; 95% CI: 1.11–2.68) as compared to patient with age-adjusted CCI ≤ 3, 2.22 times higher among patients presenting to ED with dyspnea (OR 2.22; 95% CI: 1.56–3.17) as compared to without dyspnea, 2.17 times higher among patients with NLR ≥ 3 (OR 2.17; 95% CI: 1.54–3.06), and 6.17 time higher among patients with abnormal chest radiograph findings (OR 6.17; 95% CI: 4.40–8.66). In contrast, after similar adjustment, the odds of hospitalization were 55% lower among patients with obesity, that is, a BMI ≥ 30 kg/m^2^ (OR 0.45; 95% CI: 0.32–0.64) as compared to BMI <30 and 36% lower among patients who presented to ED with fever (OR 0.64; 95% CI: 0.43–0.95) as compared to patients who reported no fever.

## 4. Discussion

Our study shows that in the early phase of the COVID-19 pandemic, 61% of patients presenting to our health system with COVID-19 infection required hospitalization, creating a significant bottleneck and overcrowding in the ED [14,15]. This early phase represented a time of limited understanding of the virus pathology, lack of targeted treatments, and no available vaccination to curtail infection spread. Patients requiring hospitalization spent more time in the ED waiting for bed availability than those discharged from the ED, further adding strain on the ED bed capacity. Predictors for higher hospitalization odds included older age and comorbidity burden, dyspnea on presentation, abnormal chest radiograph, elevated neutrophil-to-lymphocyte ratio, health insurance access, primary care provider access, and presentation from a non-home locale. Understanding these predictors could help inform targeted triage decision-making, resource allocation, and improved patient throughput in the ED.

The average time spent in the ED was higher for patients who were eventually admitted to the hospital, suggesting that hospitalized patients remained boarded in the ED prior to transfer to a hospital bed. Studies have shown that during the pandemic, while ED boarding times were rising, inpatient mortality also rose [16]. The literature has shown that high ED boarding times during the pandemic were associated with high hospital occupancy [17], which sets up a vicious cycle of strain on hospital capacity, further constraining the availability of hospital beds, airborne isolation apparatus, medical personnel, intensive care unit (ICU) resources, and ventilators [14,15,18,19].

Our study showed multimorbidity was a predictor of hospitalization (using age-adjusted CCI). This is similar to the findings from other cohorts reporting age and high morbidity burden being strong predictors of COVID-19-related hospitalization [20,21,22,23]. After adjustment for differences in sociodemographic and clinical variables, our study population did not show race as a predictor for hospitalization. The results are in contradiction with prior published studies, which have demonstrated that black race was associated with hospitalization but not higher in-hospital mortality [24,25]. Insurance access was also associated with higher odds of hospitalization in our study population, a finding concordant with the previously published literature [24]. Patients with access to a primary care provider exhibited higher odds of hospitalization, which contradicts the published literature on the efficacy of robust primary care programs in reducing COVID-19 hospitalizations. This was likely due to disrupted services of already strained primary care outpatient clinics and a shortage of telehealth during the initial surge of COVID-19 infections [26,27,28,29]. We also found that hospitalization odds were higher for patients from non-home locales, such as skilled nursing facilities and group homes, than those from home. This could have represented the limited capacity of facilities to accommodate isolation needs, staffing shortage, fear of infection breakouts, lack of vaccine availability in the early phase of the pandemic, etc. [30].

Among clinical variables, dyspnea on presentation, abnormal CXR, and elevated inflammatory marker (NLR) were associated with higher odds of hospitalization in our study. The neutrophil-to-lymphocyte ratio has been shown in multiple studies as a clinical predictor of severe COVID-19 infection and mortality [31,32]. These clinical variables could help inform triaging models in the future. Some such predictive scoring models for triaging have been published using clinical, laboratory, and radiological markers to be used in the emergency departments, which can be further bolstered with a better understanding of hospitalization predictors, as noted in this study [22,23]. Another striking finding in our study was that higher BMI was associated with lower odds of COVID-19-related hospitalization. While obesity is generally associated with an increased risk for severe COVID-19 outcomes, some studies have observed a protective effect in specific populations, a phenomenon often referred to as the “obesity paradox”. For instance, a study found that among elderly individuals, mild to moderate obesity was associated with a reduced risk of COVID-19 mortality. This counterintuitive report may represent the protective metabolic reserves and potential anti-inflammatory properties associated with increased adipose tissue in older adults [33]. Adipose tissue may act as an absorbent buffer for inflammatory cytokines, which typically surge into circulation during COVID-19 viral infection. This could be one mechanism for diminishing the inflammatory surge in obese adults. Adipokines such as leptin and adiponectin may also provide some cardioprotective reserves during critical illness, further contributing to this obesity paradox. The obesity paradox remains a topic of active debate, and as such, a true explanation is currently undetermined. Research has indicated that in specific contexts, while obesity is a transmission risk factor for SARS-CoV-2, it does not always lead to higher mortality, suggesting a nuanced and complicated relationship between obesity and COVID-19 outcomes [34]. Further research is needed to understand the underlying mechanisms contributing to this paradoxical observation.

Some limitations of this study merit consideration, including the ones innate to retrospective designs. First, this study was conducted at a single healthcare system encompassing five closely located hospitals, which may not fully represent broader demographic and clinical variations in different regions or healthcare systems. This limits the generalizability of our findings to external cohorts and healthcare settings. Second, although the models adjusted for sociodemographic and clinical variables, there may still be unmeasured confounders influencing the results. Third, the findings represent the initial surge of COVID-19, which may not apply to the subsequent virus variants. Fourth, subpopulation analyses were not possible for various disease-specific subgroups such as patients with autoimmune disorders, chronic pulmonary disorders, etc. Lastly, the data were collected from electronic medical records. So, our findings rely on the degree of accuracy of medical personnel’s clinical documentation.

## 5. Conclusions

A significant proportion of patients presenting to the ED required hospitalization during the early phase of the pandemic. The drivers of COVID-19-related hospitalizations are multifarious and complex. This study highlights predictive sociodemographic and clinical factors associated with COVID-19-related hospitalization in the emergency department. Understanding these factors may help develop triage and predictive models for future pandemic surges. Future research needs to evaluate the impact of weekend presentations and the paradoxical relationship between obesity and COVID-19 health outcomes, as these findings could guide the development of triage models to improve preparedness for future pandemics.

## Figures and Tables

**Table 1:** Characteristics study population.

Characteristics ^◊^	ED Discharges (N = 1089)	Hospital Admissions (N = 1678)	*p*-Value *
Age 55 years or greater, n (%)	280 (26)	1099 (66)	**<0.001**
BMI kg/m^2^, n (%)			**<0.001**
Less than 25	157 (14)	483 (29)	
25–29.9	240 (22)	494 (29)	
≥30	692 (64)	701 (42)	
Female, n (%)	501 (46)	762 (45)	0.79
Race			**<0.001**
White, n (%)	202 (19)	517 (32)	
African American, n (%)	289 (27)	626 (38)	
Others (Asian, American Indian, American native Hawaiian or Pacific Island), n (%)	576 (54)	498 (30)	
Ethnicity			
Hispanic or Latino, n (%)	528 (49)	379 (23)	**<0.001**
Not Hispanic or Latino, n (%)	546 (51)	1266 (77)	
Married or living with a partner, n (%)	377 (45)	538 (43)	0.28
Employment status, n (%)			**<0.001**
Employed	541 (55)	480 (31)	
Unemployed, with a disability, or unable to work	410 (42)	963 (62)	
Retired, student, homemaker	30 (3)	121 (7)	
Uninsured, n (%)	360 (36)	108 (7)	**<0.001**
No primary care physician, n (%)	518 (48)	669 (40)	**<0.001**
Median ED length of stay, minutes (IQR) **	138 (69–249)	339 (246–480)	<0.001 **
Weekend presentation to ED, n (%)	245 (23)	440 (26)	**0.027**
Current and ex-smoker, n (%)	148 (14)	427 (26)	**<0.001**
Alcohol use (current and past), n (%)	277 (26)	594 (35)	**<0.001**
Reported exposure to COVID-19, n (%)	514 (47)	486 (29)	**<0.001**
Presented to ED from nursing home, inpatient rehab, group home, adult daycare, homeless n (%)	19 (2)	397 (24)	**<0.001**
Age-adjusted Charlson Comobrbidty Index (CCI) ^¥^ > 3, n (%)	137 (13)	989 (59)	**<0.001**
Median duration of symptoms at presentation in days (IQR) **	4 (2–7)	5 (2–7)	<0.001 **

^◊^ For some patients, the variables had missing values; * Chi-Square, Fisher’s exact statistic (where at least 20% of frequencies were <5), and Unpaired *t*-test. Statistic: ** Mann–Whitney U test; ^¥^, Charlson comorbidity index (CCI)—scores of 0, 1, 2, and 3 predicting 10-year survival rates of 93%, 73%, 52%, and 45%, respectively. The boldface type indicates statistical significance. BMI, body mass index; IQR, interquartile range.

**Table 2:** Symptoms, vitals, and laboratory workup for study population at initial ED presentation.

Variables ^◊^	ED Discharges (N = 1089)	Hospital Admissions (N = 1678)	*p*-Value *
Symptoms at presentation			
Fever, n (%)	713 (66)	1024 (61)	**0.02**
Chills, n (%)	294 (27)	426 (25)	0.35
Dry cough, n (%)	616 (57)	834 (50)	**<0.001**
Productive cough, n (%)	68 (6)	158 (9)	**0.003**
Nasal congestion, n (%)	128 (12)	73 (4)	**<0.001**
Sore throat, n (%)	247 (23)	176 (11)	**<0.001**
Shortness of breath, n (%)	289 (27)	988 (59)	**<0.001**
Myalgia, n (%)	469 (43)	479 (29)	**<0.001**
Fatigue, n (%)	245 (23)	421 (25)	0.12
Headache, n (%)	214 (20)	235 (14)	**<0.001**
Loss of smell, n (%)	77 (7)	77 (5)	**0.006**
Alteration in taste sensation, n (%)	92 (8)	118 (7)	0.19
Diarrhea, n (%)	89 (8)	288 (17)	**<0.001**
Nausea, n (%)	56 (5)	125 (7)	**0.018**
Vomiting, n (%)	78 (7)	184 (11)	**0.001**
Appetite loss, n (%)	29 (3)	170 (10)	**<0.001**
Chest pain, n (%)	148 (14)	250 (15)	0.35
Abdominal pain, n (%)	47 (4)	159 (9)	**<0.001**
Generalized weakness, n (%)	54 (5)	260 (16)	**<0.001**
Syncope	22 (2)	188 (11)	**<0.001**
Dizziness, n (%)	42 (4)	94 (6)	**0.04**
Vital signs at presentation			
Systolic blood pressure, mean (SD)	134 (19)	130 (24)	**<0.001**
Diastolic blood pressure, mean (SD)	81 (12)	75 (15)	**<0.001**
Mean arterial pressure, mean (SD)	91 (16)	97 (20)	**<0.001**
Heart rate, mean (SD)	98 (13)	94 (16)	**<0.001**
Temperature in centigrade, mean (SD)	37.3 (0.64)	37.5 (0.96)	**<0.001**
Respiratory rate, mean (SD)	17 (3)	21 (7)	**<0.001**
Pulse oxygen, mean (SD)	97 (2.4)	94 (5.5)	**<0.001**
FiO_2_%, mean (SD)	21 (4.4)	28 (16.7)	**<0.001**
Laboratory and imaging workup at ED presentation			
Hemoglobin, mean (SD)	13.5 (1.9)	12.8 (2.3)	**<0.001**
White blood cell count, mean (SD)	6.2 (3.2)	8.2 (6.6)	**<0.001**
Neutrophil to lymphocyte count ratio ≥ 3, n (%)	195 (52)	1234 (75)	**<0.001**
Platelet count, mean (SD)	210 (69)	219 (93)	**0.045**
Creatinine, mean (SD)	1.15 (1.5)	1.55 (2.1)	**<0.001**
Glomerular fraction rate, mean (SD)	92 (25)	73 (34)	**<0.001**
Glucose, mean (SD)	124 (50)	149 (79)	**<0.001**
AST, mean (SD)	38 (31)	59 (131)	0.20
ALT, mean (SD)	38 (32)	45 (155)	0.32
Alkaline phosphatase, mean (SD)	83 (35)	94 (63)	**0.001**
Albumin, mean (SD)	4.2 (0.5)	3.7 (0.7)	**<0.001**
Calcium, mean (SD)	9.1 (0.5)	8.8 (0.7)	**<0.001**
CXR with abnormal read out of total CXR, n (%)	138/460 (30)	1255/1578 (79)	**0.001**

**^◊^** For some patients, the variables had missing value. * Chi-Square, Fisher’s exact statistic (where at least 20% of frequencies were <5), and Unpaired *t*-test statistic. SD, standard deviation.

**Table 3:** Clinical signs, initial workup, and admitting diagnoses for patients hospitalized with COVID-19.

Variables ^◊^	Hospital Admissions (N = 1678)
Clinical signs at presentation, n (%)	
Frail	346 (21)
Dry oral mucosa	189 (11)
Poor orodental hygiene	10 (0.6)
Conjunctival injection	9 (0.5)
Throat congestion	8 (0.5)
Tonsillar swelling	2 (0.1)
Lymphadenopathy	1 (0.06)
Respiratory distress	455 (27)
Hypoxia	421 (25)
Wheezing, rhonchi, or coarse breath sounds	285 (17)
Crackles	158 (9)
Elevated JVP	30 (1.8)
Abdominal Tenderness	116 (7)
Facial droop	6 (0.4)
Angle of mouth deviation	1 (0.06)
Motor weakness	37 (2)
Sensory loss	9 (0.5)
Tremors	19 (1.1)
Edema	86 (5)
Unresponsive	61 (4)
Laboratory workup at presentation	
Serum ferritin, median (IQR)	641.5 (310–1189)
CRP, median (IQR)	11.3 (4.4–34.9)
Erythrocyte sedimentation rate, mean (SD)	60 (35)
LDH, median (IQR)	353.5 (258–503)
Positive troponin, n (%)	302 (18)
D-dimer, median (IQR)	0.99 (0.53–2.08)
IL6 checked n (%)	648 (39)
IL6, median (IQR)	60.6 (24.1–153.6)
Procalcitonin checked n (%)	859 (51)
Procalcitonin high risk, n (%)	241 (14)
Positive blood culture, n (%)	86 (5)
Urine culture positive, n (%)	143 (9)
Sputum culture positive, n (%)	32 (2)
Imaging workup at presentation, n (%)	
Duplex studies conducted at extremities to r/o DVT	60 (4)
CT head and neck with abnormality	46 (2.7)
CT Chest with abnormality	382 (23)
CTA chest with abnormality	30 (2)
CT abdomen pelvis with abnormality	89 (5)
Echo with abnormal read	52 (3)
MRI brain with abnormal read	11/26 (0.7)
Top 10 Primary diagnoses at admission, n (%)	
Acute respiratory failure	667 (40)
Pneumonia	992 (59)
Pulmonary embolism	21 (1.2)
Congestive heart failure	41 (2.4)
Sepsis	148 (9)
COPD exacerbation	34 (2)
Deep venous thrombosis	9 (0.5)
Cerebrovascular event	6 (0.4)
Acute renal failure	142 (8)
Acute bronchitis	101 (6)

**^◊^** For some patients, the variables had missing values.

**Table 4:** Predictors of hospitalization for patients presenting to ED with COVID-19 positive infection.

Risk Factors Analysis	Odds Ratio (95% CI)	
Socioeconomic predictors for hospitalization	Unadjusted Model *	Adjusted Model **
Age > 55 years	**5.48 (4.63–6.50)**	**1.59 (1.24–2.05)**
Unemployed with a disability or unable to work, retired, homemaker, or student	**2.78 (2.35–3.28)**	**1.33 (1.07–1.66)**
Health insurance	**7.67 (6.07–9.69)**	**3.61 (2.64–4.94)**
Access to primary care	**1.37 (1.17–1.60)**	**1.95 (1.58–2.40)**
Alcohol use (prior and current)	**1.61 (1.36–1.90)**	**1.58 (1.26–1.99)**
Presented to ED from nursing home, inpatient rehab, group home, adult daycare, or homeless	**17.5 (10.9–27.9)**	**4.58 (2.73–7.67)**
Obesity (BMI ≥ 30 vs. <30 kg/m^2^)	**0.41 (0.35–0.48)**	**0.58 (0.47–0.71)**
Weekend presentation to ED	**1.22 (1.02–1.46)**	**1.34 (1.06–1.71)**
Age-adjusted Charlson Comobrbidty Index (CCI) > 3	**9.97 (8.14–12.2)**	**3.84 (2.88–5.10)**
Symptoms and lab predictors for hospitalization	Unadjusted Model *	Adjusted Model ***
Fever	**0.83 (0.70–0.97)**	**0.64 (0.47–0.89)**
Shortness of breath	**3.96 (3.36–4.68)**	**1.93 (1.43–2.60)**
Myalgia	**0.53 (0.45–0.62)**	**0.70 (0.51–0.95)**
Chest pain	1.11 (0.89–1.37)	**0.60 (0.42–0.85)**
Neutrophil to lymphocyte count ratio ≥ 3	**2.89 (2.29–3.64)**	**2.34 (1.76–3.12)**
CXR with abnormal read	**9.07 (7.18–11.5)**	**6.16 (4.62–8.21)**
Full adjusted model for both socioeconomic, presenting symptoms, and initial lab work		Adjusted Model ****
Health insurance		**3.44 (1.98–6.0)**
Access to primary care		**1.85 (1.33–2.59)**
Obesity (BMI ≥ 30 vs. <30 kg/m^2^)		**0.45 (0.32–0.64)**
Presented to ED from nursing home, inpatient rehab, group home, adult daycare, or homeless		**4.04 (1.93–8.47)**
Age-adjusted Charlson Comobrbidty Index (CCI) >3		**1.72 (1.11–2.68)**
Fever		**0.64 (0.43–0.95)**
Shortness of breath		**2.22 (1.56–3.17)**
Neutrophil to lymphocyte count ratio ≥ 3		**2.17 (1.54–3.06)**
CXR with abnormal read		**6.17 (4.40–8.66)**

* Unadjusted model for individual variables. ** Adjusted model for socioeconomic risk factors thought to influence hospitalization (age, gender, race, ethnicity, marital status, employment status, having insurance, having a primary care provider, smoking status, alcohol use, obesity, weekend admission, presenting from nursing home/inpatient rehab/group home/adult daycare or homeless, and Charlson comorbidity index). *** Adjusted model for clinical symptoms, initial laboratory and imaging workup thought to influence hospitalization (fever, cough, sore throat, shortness of breath, myalgia, fatigue, headache, diarrhea, vomiting, chest pain, generalized weakness, neutrophil to lymphocyte ration, and chest X-ray). **** Adjusted model for both above-mentioned models including socioeconomic risk factors (age, gender, race, ethnicity, marital status, employment status, having insurance, having a primary care provider, smoking status, alcohol use, obesity, weekend admission, presenting from nursing home/inpatient rehab/group home/adult daycare or homeless, and Charlson comorbidity index) and clinical symptoms, initial laboratory and imaging workup (fever, cough, sore throat, shortness of breath, myalgia, fatigue, headache, diarrhea, vomiting, chest pain, generalized weakness, neutrophil to lymphocyte ration, and chest X-ray). The boldface type indicates statistical significance. BMI, body mass index; SD, standard deviation; CI, confidence interval.

## Data Availability

The original contributions presented in this study are included in the article. Further inquiries can be directed to the corresponding author.
